# Pacific climate variability and the possible impact on global surface CO2 flux

**DOI:** 10.1186/1750-0680-6-8

**Published:** 2011-10-08

**Authors:** Hideki Okajima, Michio Kawamiya

**Affiliations:** 1 Research Institute for Global Change, Japan Agency for Marine-Earth Science and Technology, Yokohama 236-0001, Japan

## Abstract

**Background:**

Climate variability modifies both oceanic and terrestrial surface CO2 flux. Using observed/assimilated data sets, earlier studies have shown that tropical oceanic climate variability has strong impacts on the land surface temperature and soil moisture, and that there is a negative correlation between the oceanic and terrestrial CO2 fluxes. However, these data sets only cover less than the most recent 20 years and are insufficient for identifying decadal and longer periodic variabilities. To investigate possible impacts of interannual to interdecadal climate variability on CO2 flux exchange, the last 125 years of an earth system model (ESM) control run are examined.

**Results:**

Global integration of the terrestrial CO2 flux anomaly shows variation much greater in amplitude and longer in periodic timescale than the oceanic flux. The terrestrial CO2 flux anomaly correlates negatively with the oceanic flux in some periods, but positively in others, as the periodic timescale is different between the two variables. To determine the spatial pattern of the variability, a series of composite analyses are performed. The results show that the oceanic CO2 flux variability peaks when the eastern tropical Pacific has a large sea surface temperature anomaly (SSTA). By contrast, the terrestrial CO2 flux variability peaks when the SSTA appears in the central tropical Pacific. The former pattern of variability resembles the ENSO-mode and the latter the ENSO-modoki^1^.

**Conclusions:**

Our results imply that the oceanic and terrestrial CO2 flux anomalies may correlate either positively or negatively depending on the relative phase of these two modes in the tropical Pacific.

## Background

The Pacific Ocean is the largest oceanic domain on Earth and has the greatest impact of all ocean basins on climate variabilities on both a global and regional scale. One of the most dominant climatic phenomena on an interannual time scale is El Nino Southern Oscillation (ENSO). The Pacific ENSO has largest variance along the equator because it is excited by the Bjerknes feedback [1]. For example, the enhanced zonal SST gradient makes the trade winds stronger and the thermocline tilt steeper, and hence the initial zonal SST gradient anomaly is further enhanced. Thus, the anomalous zonal SST gradient, trade winds, and thermocline tilt are closely connected at the equator in such a way that the initial perturbations grow rapidly through this feedback process. As a climatic impact, the zonal and vertical atmospheric circulation, the so-called Walker cell, is strengthened over the equatorial Pacific and brings anomalous high (low) pressure systems to the east (west) of the Pacific, resulting in Peruvian droughts and Indonesian floods. In the meridional direction, the anomalous tropical SST and trade winds also modify the atmospheric circulation on a global scale by displacing the foot of the Hadley cell and changing the stationary wave pattern [2,3]. Therefore, the tropical SST anomaly can impact on climate not only in the tropics but also remotely at higher latitudes. The ENSO spectra has multiple peaks around the quasi-biennial or quasi-quadrennial frequency depending on the coupling parameter, shown by many model studies during the Tropical Ocean-Global Atmosphere (TOGA) program (refer to review article [4]).

Other than the ENSO, several Pacific variabilities have been proposed. The Pacific Decadal Oscillation (PDO) has a long-lived ENSO-like climate variability pattern in the Pacific [5,6]. Compared to ENSO, the PDO events have maximum variance in the northeastern Pacific rather than in the tropics, with a timescale of 20 to 30 years. Some studies have shown that the PDO in the 20th century had multi-decadal modes, one with periods of 15 to 25 years, and the other of 50 to 70 years [7]. These decadal climate variabilities were first found through Alaskan salmon production research and hence are closely related to the marine ecosystem productivity in the basin-wide North Pacific. More recent studies suggest that yet another Pacific climate variability dominates the SST anomaly around the central tropical Pacific near the date line. This phenomena is variously referred to as either ENSO-modoki [8-10], warm-pool ENSO [11], or central-Pacific ENSO [12,13], and features basin-wide and decadal-scale variability in an ocean and atmosphere coupled system. Some studies further point out that global warming is related to the spatio-temporal modulation of the anomalous event [12,13]. These decadal to interdecadal modes have been investigated in relation to the climatic regime shift in the late 1970s or recent unusual tropical variability, although their mechanism is still unclear.

These interannual and longer-term climate variabilities also modify both the ocean-atmosphere CO2 flux and the land-atmosphere CO2 flux by changing the oceanic and terrestrial biogeochemical cycles. Using observation and assimilated data sets, earlier studies have shown that tropical oceanic climate variability has strong impacts on the land surface temperature and soil moisture, and there is a negative correlation between oceanic and terrestrial CO2 fluxes [14,15]. However, these data sets only cover less than the most recent 20 years and are insufficient for identifying decadal and longer periodic variabilities. Zeng et al. [16] performed simulations for the twentieth century by giving observed SST anomalies to an atmospheric general circulation model (AGCM) with a sophisticated land ecosystem model. They showed how ENSO impacts on the CO2 flux over tropical land regions, which accounts for a large portion of the global interannual CO2 variability. During the El Nino phase, for instance, most of the tropical land regions experience anomalous soil temperature warming with less precipitation, resulting in a large terrestrial carbon release to the atmosphere due to increased soil respiration and decreased net primary production. However, their experiments lack feedback processes between the ocean and atmosphere.

The present study examines the relations between Pacific climate variabilities and anomalies of the surface CO2 exchange by using a coupled climate-carbon cycle GCM. We conduct a simple control experiment to show that the climate variabilities in the tropical Pacific Ocean play an important role in modifying both oceanic and terrestrial CO2 flux at a global scale. We choose to focus on the tropical Pacific variabilities because of their climatic importance.

## Methods

The Earth System Model (ESM) used in the present study is an ocean-atmosphere-land coupled general circulation model that includes physical and biogeochemical processes. It has been jointly developed at the Atmosphere Ocean Research Institute (formerly known as Center for Climate System Research) of the University of Tokyo, the National Institute for Environmental Studies, and the Japan Agency for Marine-Earth Science and Technology.

The atmospheric component is a global spectral model with a resolution of T42 in the horizontal and 20 sigma levels in the vertical. The land surface component, which describes heat and water exchange, has the same resolution as the atmospheric component in the horizontal and six to nine variable layers in the vertical, depending on the snow amount. The ocean component has a finer horizontal resolution: the longitudinal grid spacing is 1.4 degrees and the meridional grid intervals vary from 0.5 degrees at the equator to 1.7 degrees near the polar regions. The vertical resolution is 44 levels in sigma-z hybrid coordinate system, including eight sigma-layers near the surface and one bottom boundary layer [17]. Both the land and ocean component feature carbon-cycle processes. The land component has five compartments of carbon storage and 20 types of vegetation [18]. The ocean component incorporates a simple biogeochemical process: Nitrogen-Phytoplankton-Zooplankton-Detritus, which reasonably simulates the seasonal excursion of oceanic biological activities at a basin-wide scale [19]. See Kawamiya et al. [20] and Yoshikawa et al. [21] for details. The model results are also found in an article by the Coupled Carbon Cycle Climate Model Intercomparison Project [22] and in the latest report by the Intergovernmental Panel on Climate Change [23].

In the model, the atmosphere and ocean components exchange surface fluxes every three hours. We firstly spin up the model with the observed monthly climatology as the boundary condition. During the spin-up, the atmospheric CO2 concentration is fixed to a constant preindustrial value of 285 ppmv. The globally integrated CO2 fluxes between the atmosphere and land/ocean reach a quasi-steady state after about 280 model years, and then the model run is extended for another 250 years for climate simulation. This 280-year period may be insufficient for complete spin-up for the global terrestrial and oceanic carbon cycle, but is still long enough to drive the model to a quasi-steady state, i.e., the global net atmosphere-ocean CO2 exchange becomes sufficiently small compared to its interannual variability. Due to limited computational resources, we do not perform thousands of years of spin-up. The results for the surface CO2 flux analysis should be basically the same for either 280 years or longer periods of spin-up. Immediately following the spin-up experiment, the CO2 concentration is allowed to vary and the model year count begins. Results for the last 125 years of the 250-year run are analyzed in this study. As the aim of this study is to analyze the relation between climate variability and CO2 flux anomaly, we focus on the spatio-temporal structure of simulated surface temperature and surface CO2 flux.

**Figure 1:**
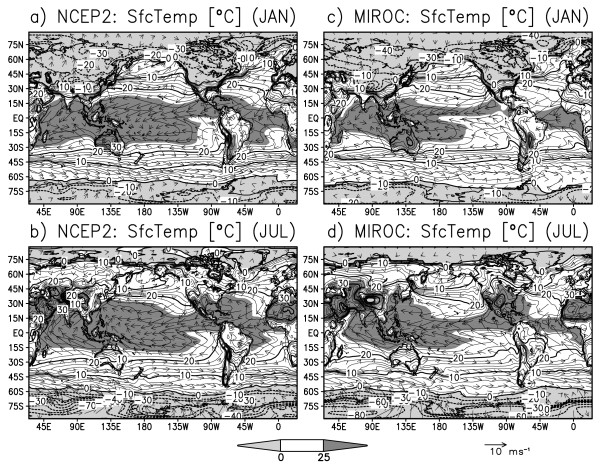
**Global distribution of surface temperature (°C) and surface wind (m s^-1^) in monthly climatology: NCEP2 reanalysis **[26]**in a) January and b) July, and ESM simulation in c) January and d) July**. Contour interval is 2°C for positive values and 10°C for negative values, with light shading *<*0°C and dark shading *>*25°C.

Prior to discussing the relation between interannual climate variability and CO2 flux anomaly, we validate the model output in terms of both annual mean and seasonal cycle. Figure 1 displays reanalyzed and simulated sea surface temperature (SST) and land surface temperature, in January and July climatology. All panels show that maximum SSTs are found over the tropical oceans: warm pools in the eastern Indian Ocean and western Pacific, and northerly displaced intertropical convergence zone in the central to eastern Pacific, seen as a high-temperature belt. Minimum SSTs are found in the polar regions. In the North Atlantic Ocean, the SST is warmer than other regions in similar latitudes, reflecting the well-resolved nature of the Atlantic meridional circulation. On the continents, surface temperature is warmest in the subtropical desert regions such as the West Sahel and Arabian peninsula, and coolest over Antarctica. Other cold spots are found over the Tibetan Plateau and Greenland ice-sheets. As for seasonality, the wind directions are reversed over the Asian, Australian, and African monsoon regions, indicating a realistically simulated ocean-land thermal contrast in the tropics and mid-latitudes.

Overall, the ocean surface has less seasonal variance compared to the land surface because of the difference of the heat capacity between sea water and land soil. The continental seasonal variance is smaller at lower latitudes and greater at higher latitudes. In contrast, the oceanic seasonal variance is large in the sea-ice regions, equatorial Pacific, and mid-latitudes in the western boundary current regions. For a detailed description of model performance, the reader is referred to other publications (e.g., "MIROC3.2 medres" in Randall et al. [23]).

**Figure 2:**
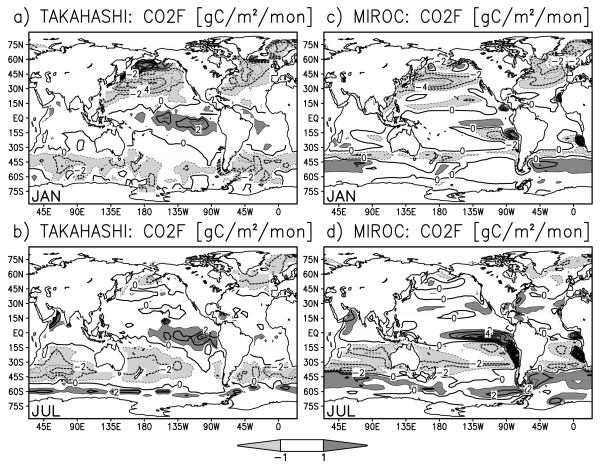
**Same as Fig. 1 but for ocean surface CO2 flux (gram carbon m^-2 ^month^-1^) by Takahashi et al.'s observation **[27]**(a,b) and ESM simulation (c,d)**.

**Figure 3:**
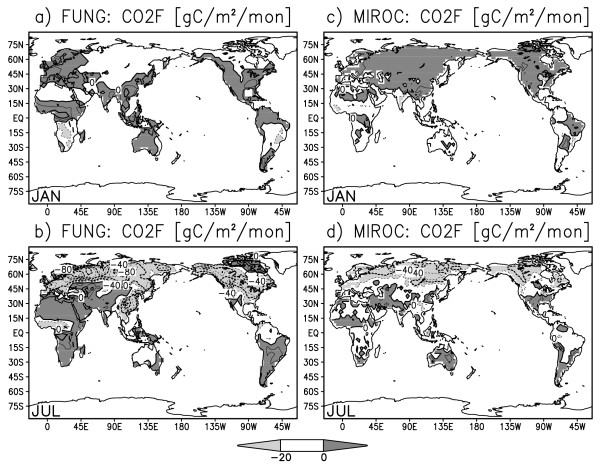
**Same as Fig. 2 but for land surface CO2 flux (gram carbon m^-2 ^month^-1^) by Fung et al.'s estimation **[28]**(a,b) and ESM simulation (c,d)**.

**Figure 4:**
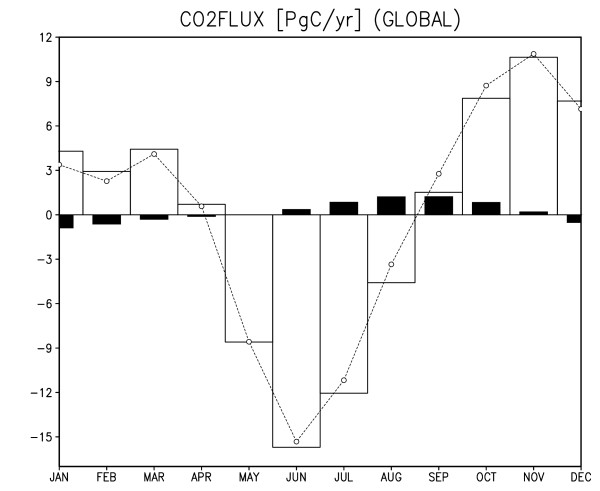
**Monthly climatology of globally integrated CO2 flux (PgC year^-1^) in the simulation**. Black (white) bars are for oceanic (terrestrial) CO2 fluxes. Short dashed line with open circles is the sum of oceanic and terrestrial CO2 fluxes. Positive values indicate that the fluxes are upward (to the atmosphere).

Figure 2 shows observed and simulated surface CO2 flux in the January and July climatology. The CO2 flux is positive (i.e., outgassing from the ocean to the atmosphere) in the equatorial eastern Pacific and Antarctic circumpolar regions where CO2-rich deep water upwells to the surface. On the other hand, the CO2 flux is negative (i.e., uptaking from the atmosphere to the ocean) in the North Atlantic where the sea surface water sinks to form North Atlantic deep water. The globally averaged oceanic CO2 flux is negative during the boreal winter to spring and becomes positive during the boreal summer to fall (black bars in Figure 4). This seasonality well coincides with the seasonal development of the Pacific cold tongue: as the equatorial and coastal upwelling become stronger in boreal summer, deep sea water with abundant dissolved inorganic carbon (DIC) is advected to the ocean surface and more CO2 is released to the atmosphere. SST cooling would lessen CO2 emission because colder sea water has greater gas solubility, but for strong upwelling regions, the difference in DIC concentration is so large between the surface and deep ocean that the changes in SST and solubility are less influential. For off-equatorial and off-coastal regions, the SST anomaly has a direct effect on the CO2 flux through the change in oceanic gas solubility. Figure 3 illustrates the monthly climatology of the estimated and simulated CO2 flux over terrestrial regions. The terrestrial CO2 flux is positive in September-March and turns negative in May-August, that is, the land in total absorbs CO2 during the boreal spring to summer and emits CO2 during the boreal fall to winter (please also see white bars in Figure 4). In the boreal spring and summer, plants in the Northern Hemisphere exhibits vigorous photosynthesis, while in fall and winter, leaves fall and litter accumulates.

Another reason for winter terrestrial net outgassing is that heterotrophic respiration is greater than gross primary production in winter. As the majority of land areas lie in the Northern Hemisphere, these dominate the seasonal modulation of terrestrial CO2 flux. Thus, there is a phase difference of about a quarter-period between the oceanic and terrestrial CO2 flux in the annual cycle (Figure 4). The terrestrial CO2 flux has about an order of magnitude larger amplitude than the oceanic flux, in good agreement with assimilated data [15].

The seasonal variation, in Figure 2 and 3, is greater over the continents than over the ocean by an order of magnitude except for areas covered by ice or snow. The oceanic CO2 variation is especially large in the eastern tropical Pacific and eastern tropical Atlantic, where the equatorial and coastal upwelling has strong variation both on seasonal and interannual time scales [14]. The terrestrial CO2 variation strongly depends on vegetation type and is generally large in the tropical savanna regions and mid-latitude crop fields, and small over deserts and ice sheets. The horizontal distribution of net CO2 flux over the continents is rather scattered and no systematic spatial pattern is discerned.

## Results and discussion

In the previous section, the model climatology has been displayed along with the reanalysis/observation to compare and validate the model performance. Now we shift our focus to the climate variability in order to examine the relations between interannual variabilities in surface temperature and surface CO2 flux.

**Figure 5:**
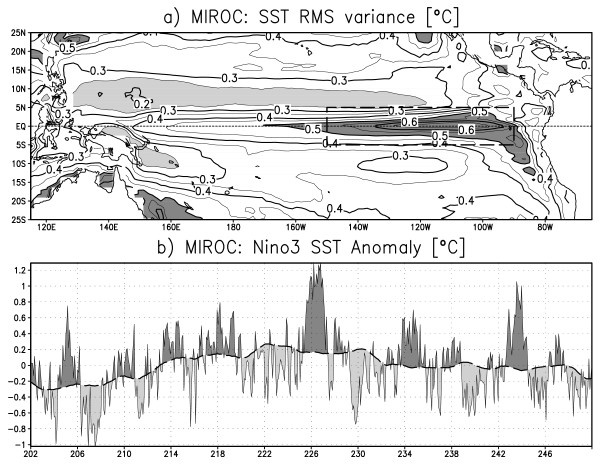
**a) SST root-mean-square variance (contour interval of 0.5°C with light shading *<*0.25°C and dark shading *>*0.5°C)**. b) Time series of Nino3 area mean SST anomaly (°C). The thin solid line is the monthly anomaly and the thick dashed line is the 120-month running mean. The annual cycle is removed before analysis and the result for the last 48 years of the model run is depicted for figure clarity.

Figure 5a shows the SST root-mean square variance in the tropical Pacific Ocean. The maximum variance is found in the eastern Pacific along the equator and along through the South American coast, indicating that the most dominant SST variability is trapped along the eastern equatorial and coastal regions. ENSO is realistically simulated in terms of spatial variability pattern although the maximum variance is smaller than observed [24,25]. Figure 5b shows the time series of Nino3 area averaged SST anomaly. The amplitude of the SST anomaly ranges between 0.4 and 1.4°C. The anomalous SST changes its sign on a time scale of 2-5 years. The model ENSO is weaker than real ENSO, but its horizontal distribution and characteristic frequency are well simulated.

**Figure 6:**
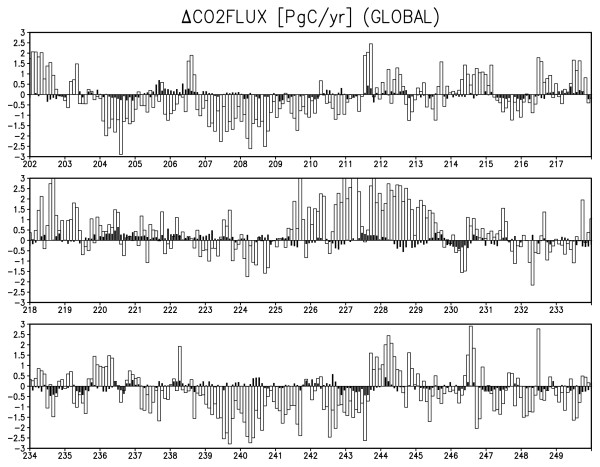
**Time series of globally integrated CO2 flux anomalies (PgC year^-1^) in the simulation**. Black (white) bars are for monthly oceanic (terrestrial) CO2 flux anomalies. Positive values indicate that the fluxes are upward (to the atmosphere). Horizontal axis is model years after spin-up. The annual cycle is removed before analysis and the result for the last 48 years is depicted for figure clarity.

Figure 6 shows the time series of globally integrated CO2 flux anomaly from the simulation. Similar to the annual cycle, the terrestrial CO2 flux anomaly has an amplitude about an order of magnitude greater than the oceanic flux on an interannual timescale. However, the periodic time scale seems to be different between oceanic and terrestrial CO2 flux. The oceanic CO2 flux has about 2-4 year periodic variability, but the terrestrial CO2 flux appears to vary over decadal or longer time scales. Therefore, in some years the oceanic and terrestrial CO2 flux anomalies have the same sign, whereas in other years they have opposite signs. For example, in Figure 6, the terrestrial CO2 flux anomaly is broadly negative for model years from the mid 200s until the early 210s, turns positive for the mid 210s and late 220s, and again turns negative from the late 230s until the early 240s. Meanwhile, the oceanic CO2 flux anomaly changes its sign more frequently, and therefore anomalies with the same sign are found for model years 204, 208-209, 215-217, 227, 243, and 247, but those with opposite signs are found for model years 206-207, 214, 228, and 239-242. This result implies that the terrestrial CO2 flux is influenced not only by ENSO variability but also by other independent modes, because ENSO variability should to drive the oceanic and terrestrial CO2 flux to negatively correlate [16].

**Figure 7:**
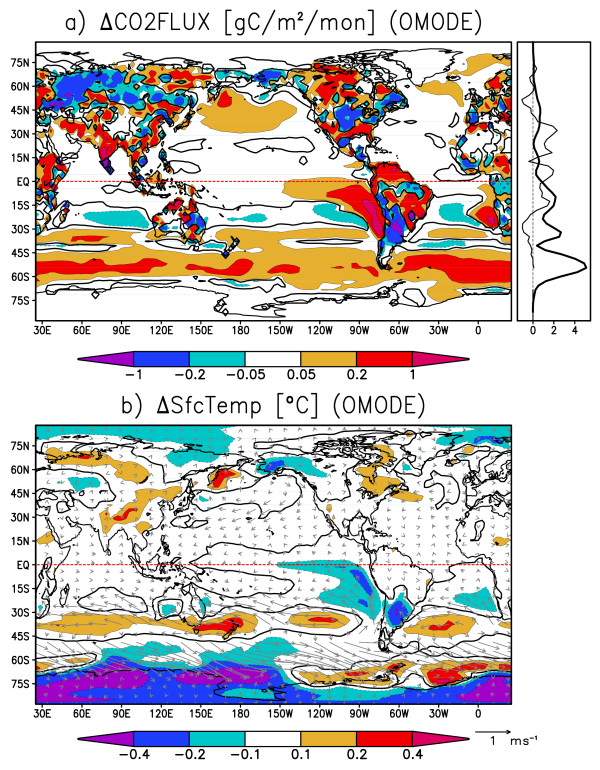
**Composite maps for the oceanic mode: a) CO2 flux (contours and shades, gram carbon m^-2 ^month^-1^), and b) sea surface temperature (contours and shades, °C) and surface winds (vectors in m s^-1^)**. The composite is for months when the globally integrated oceanic CO2 flux anomaly exceeds 0.15 PgC year^-1 ^or falls below -0.15 PgC year^-1^. Thick black contours are zero-lines. The line plots next to the panel (a) indicate the zonally integrated anomalies in the composite: oceanic (thick line, PgC year^-1^) and terrestrial (thin) CO2 fluxes.

Table 1 shows the contributions of terrestrial and oceanic latitudinal bands to the global CO2 flux variance. The greatest variance is found in the tropical terrestrial regions, which account for nearly half of the global CO2 flux variance. The second greatest variance is in the northern extratropical terrestrial regions, and the third greatest variance is seen in the tropical oceanic regions, which accounts for about a half of the global oceanic CO2 flux variance. Since the tropical regions have the greatest CO2 variance for both land and ocean, we hereafter focus on how the tropical climate variabilities will modulate the global CO2 fluxes. The extratropical continents can be influenced by the teleconnections from tropical climate variabilities. To determine the spatial pattern of variability, we perform composite analysis by taking the global CO2 flux anomaly as an index. Figures 7a and 7b are the composite maps for the oceanic mode when the global oceanic CO2 flux anomaly is negative. In Figure 7a, the oceanic CO2 flux is positive almost everywhere, having peaks off the Peruvian and Namibian coasts. The terrestrial CO2 flux is positive over northeastern Africa, southern Asia, and northern South America, while negative over central Eurasia, northwestern North America, and southern South America. Fluxes from these regions cancel each other, making the global terrestrial CO2 flux nearly zero. In Figure 7b, the characteristic feature in the tropical oceans is the cold SST anomaly along the Peruvian coast and equatorial Pacific, accompanied by anomalous easterly winds, indicative of Bjerknes feedback, which plays a key role in the ENSO variability. The Southern Ocean shows anomalous outgassing along with enhanced westerlies, possibly related to the Southern Annular Mode, while the SST anomaly is correlated to neither the CO2 flux nor surface winds, and its relation to Pacific ENSO is unclear. For the ocean, we focus on the tropics because of its greatest contribution to global CO2 flux variabilities.

**Table 1:** The CO2 flux variance (PgC year^-^^1^) by region, and percentage of the global modulation from the model result.

Region	LAND	OCEAN
30N-90N	4.03 (36.32%)	0.35 (3.17%)
30S-30N	5.19 (46.70%)	0.79 (7.11%)
90S-30S	0.16 (1.46%)	0.58 (5.24%)

TOTAL	9.38 (84.48%)	1.72 (15.52%)

**Figure 8:**
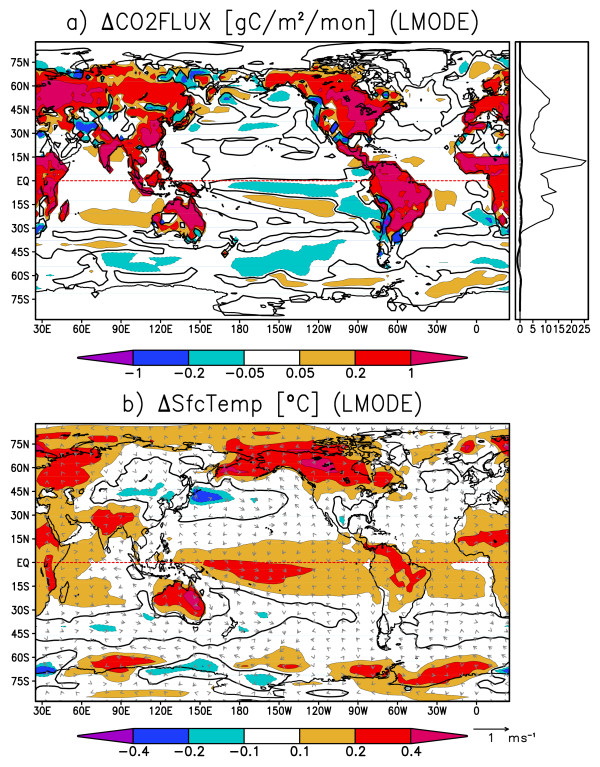
**Same as Fig. 7 but for the terrestrial mode**. The composite is for months when the globally integrated terrestrial CO2 flux anomaly exceeds 0.6 PgC year^-1 ^or falls below - 0.6 PgC year^-1^.

Figures 8a and 8b are the composite maps for the land mode when the global terrestrial CO2 flux is anomalously positive. In Figure 8a, terrestrial CO2 flux is mostly positive over continents. The oceanic CO2 flux is small compared to the terrestrial flux, and its sign is not uniform and roughly cancels out when globally integrated. In Figure 8b, a warm SST anomaly is found in the tropical Pacific centered around the date line, and its meridional distribution is not confined to the oceanic equatorial radius of deformation, a few degrees north and south of the equator. Also, strong outgassing anomalies accompanied by warm surface temperature anomalies are found over all of the tropical continents. Comparison between Figure 7b and Figure 8b shows that the terrestrial mode is driven by a mode somewhat different from normal ENSO. Instead, the spatio-temporal feature of this variability resembles that of ENSO-modoki, as a SST anomaly appears in the central tropical Pacific with decadal-scale variability [8,9].

Before concluding that the oceanic and terrestrial CO2 flux are influenced by ENSO and ENSO-modoki, respectively, we confirm that those modes in fact modify the surface CO2 flux, by taking composites in a reverse manner. Here, we use area-averaged SST anomalies to calculate the indices of each mode as follows: The Nino3 index is defined by the SST anomaly in the eastern equatorial Pacific (150W-90W, 4S-4N), and ENSO-modoki index (*EMI*) by the difference of central tropical Pacific (*CP*: 165E-140W, 10S-10N) and an average of eastern (*EP*: 110W-70W, 15S-5N) and western (*WP*: 125E-145E, 10S-20N) tropical Pacific SST anomalies (e.g. *EMI *= *CP *- (*EP *+ *WP*)*/*2) [8].

**Figure 9:**
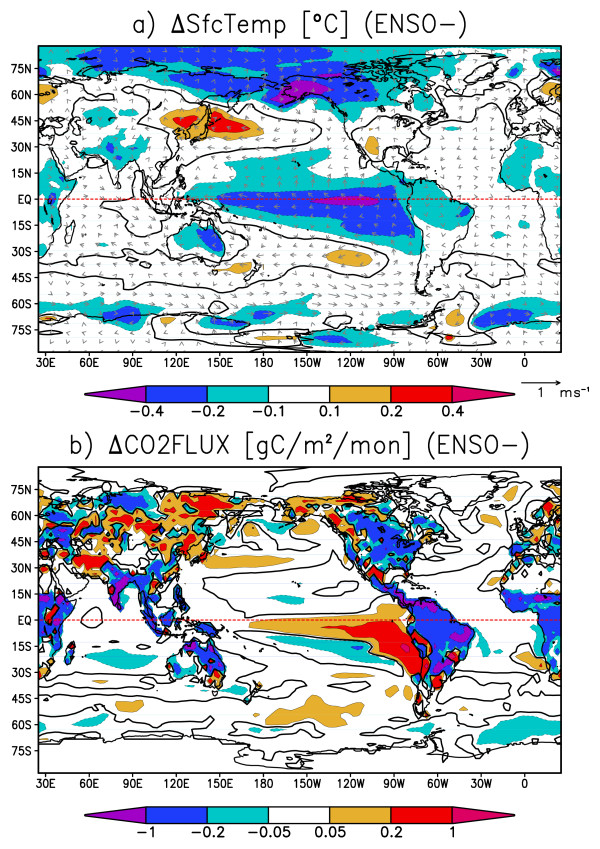
**Composite maps for the ENSO mode: a) sea surface temperature (contours and shades, °C) and surface winds (vectors in m s^-1^), and b) CO2 flux (contours and shades, gram carbon m^-2 ^month^-1^)**. The composite is for months when the ENSO indices exceed 0.05°C or falls below 0.05°C. Thick black contours are zero-lines.

**Figure 10:**
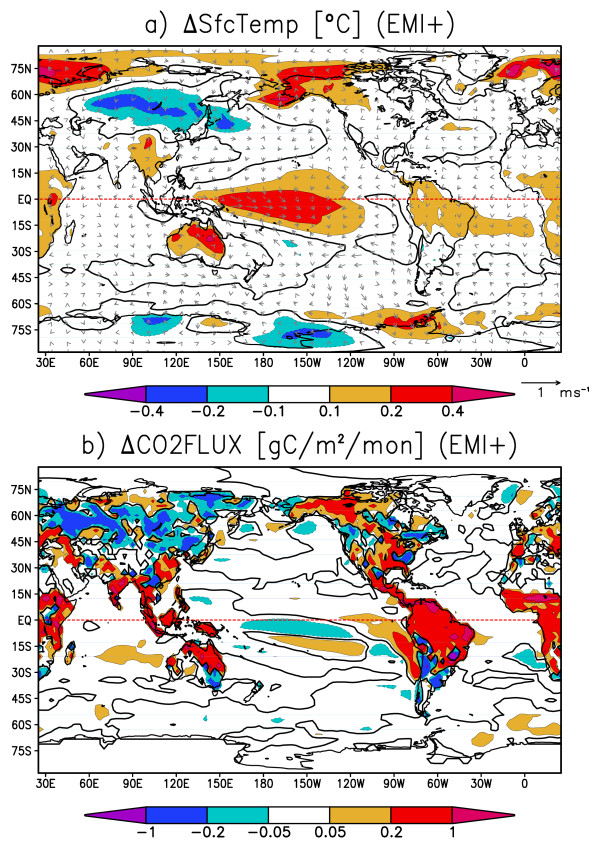
**Same as Fig. 9 but for ENSO-modoki**.

Figure 9a and 9b are the composite maps for the ENSO mode, when the Nino3 SST anomaly is positive. In Figure 9a, strong negative SST anomalies are found in the eastern Pacific along the Peruvian coast and along the equatorial Pacific. Tropical trade winds are strengthened by the easterly anomalous equatorial winds along with the SST anomaly. On the continents, cold anomalies are found over the Amazon and northeastern Australia, and warm anomalies lie over eastern Eurasia and the center of North America. Figure 9b shows the corresponding CO2 flux anomaly when Nino3 SST anomaly is positive. The CO2 flux anomalies in the eastern and equatorial Pacific are strongly positive, indicating that the CO2 emissions there are greatly enhanced by stronger equatorial upwelling. Thus, the SST and CO2 flux are negatively correlated in the tropical oceans. This result is in good agreement with recent studies [14,15]. On the other hand, terrestrial CO2 flux anomalies in many locations are positively correlated with the surface temperature primarily due to the changes in soil carbon storage. Negative correlations are found between CO2 flux and surface temperature over North America, where vegetation carbon storage has a larger contribution to CO2 variability [21]. Therefore, the CO2 flux anomaly caused by ENSO (Figure 9b) shows a certain level of resemblance to the oceanic-mode-origin CO2 flux anomaly (Figure 7a) in that strong CO2 flux anomalies prevail in the equatorial and coastal upwelling regions in the eastern Pacific. Surface temperature, like CO2 flux, shows maximum anomalies at the equator and along the Peruvian coast accompanied by the surface wind anomalies enhanced by the Bjerknes feedback (Figure 7b and 9a). Figure 10a and 10b are the composite maps for the ENSO-modoki mode when the ENSO-modoki index, as defined by Ashok et al. [8], is anomalously positive. In the surface temperature composite map (Figure 10a), a warm SST anomaly is formed in the central tropical Pacific. Unlike the ENSO pattern, this SST anomaly does not reach the eastern edge of the Pacific basin and is characteristic of ENSO-modoki. The terrestrial CO2 flux anomalies are strongly positive over tropical land masses, such as the Amazon, southeastern Asia, and Australia. The oceanic CO2 flux anomalies are not strong and either positive or negative and roughly cancel out when globally integrated. Thus, both the CO2 flux anomaly caused by ENSO-modoki (Figure 10b) and the terrestrial-mode CO2 flux anomaly (Figure 8a) show similarities, including strong anomalies over the tropical continents, and weak and randomly scattered anomalies over the oceans. ENSO-modoki is positively, though weakly, correlated with the oceanic CO2 flux, as opposed to ENSO. On the other hand, both ENSO and ENSO-modoki are positively correlated with terrestrial CO2 flux with moderate correlation coefficients. The oceanic and terrestrial CO2 fluxes thus have different relations with climatic modes.

**Figure 11:**
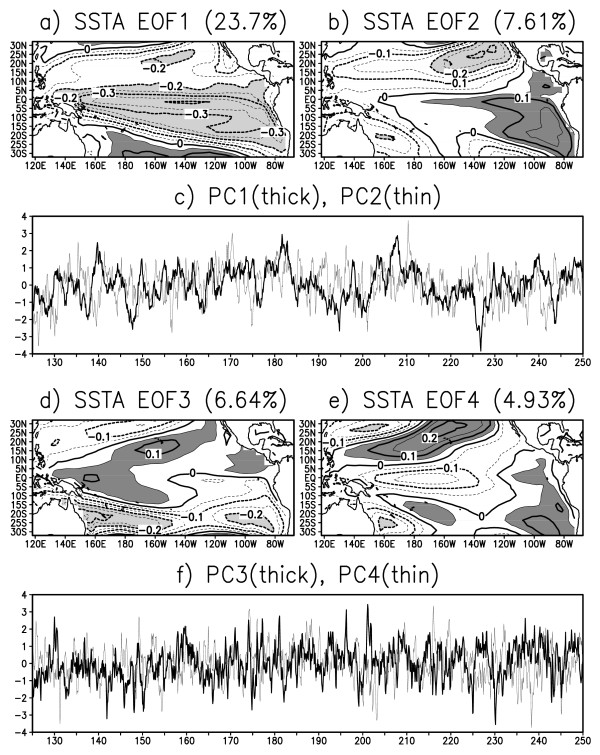
**Leading modes in the ESM**. Empirical orthogonal function analysis is applied for the Pacific SST anomaly: spatial patterns (contour intervals are 0.05 with light shading *<*-0.2 and dark shading *>*0.05) and explained variances (percentage above each panel) for a) the first mode, b) second mode, d) third mode, and e) forth mode, and time series of principal components for c) the first and second mode, and f) third and forth mode (thick and thin lines, respectively).

To examine the characteristic modes in the ESM, an empirical orthogonal function analysis is performed. Figure 11 shows the leading modes in the simulated Pacific climate. The horizontal distribution of the first mode resembles a mixture of ENSO and ENSO-modoki, as the SST anomaly is the greatest on the equator but with the maximum amplitude in the central Pacific, not in the eastern Pacific. The principal component curve also suggests that the period of the first mode is 10 years or longer. The second mode has features rather similar to ENSO as the spatial pattern shows a maximum along the equator to eastern coastal regions off Central and South America, with a periodic timescale of 2-4 years. However, its amplitude is about 50% smaller than actual ENSO. The third and forth modes seem to be multi-decadal modes as they have much smaller amplitudes and longer timescales [7]. The SST anomalies stretch westward toward the equator until the Pacific warm pool, showing a characteristic pattern of PDO and multi-decadal modes but their principal components are not confined to a decadal period. Thus, the simulated ENSO is much weaker than real ENSO, allowing other modes to emerge more clearly than in reality. This might be why ENSO-modoki appears as a mixed mode in the earlier modes.

## Conclusions

We have carried out experiments with a climate-carbon cycle coupled GCM to investigate possible impacts of interannual to interdecadal climate variability upon surface CO2 flux. The model climatology bears features consistent with earlier studies using uncoupled GCMs or assimilated data sets. The seasonal excursion of terrestrial and oceanic CO2 flux anomalies proceeds in a correlated manner with phase difference of a quarter period. On the interannual timescale, by contrast, the CO2 flux anomalies are not always negatively correlated, as the terrestrial CO2 flux variability has a longer period than the oceanic flux. A series of composite analyses shows that the oceanic CO2 flux anomalies are associated with ENSO variability, while the terrestrial CO2 flux anomalies are associated with ENSO-modoki. Our results imply that the oceanic and terrestrial CO2 flux anomalies may correlate either positively or negatively depending on the relative phase of the two climate modes in the tropical Pacific. Earlier studies investigated either less than 20 years of assimilated data set or historical ENSO cases in an AGCM simulation. Our findings provide new insight via successful simulation and analysis of longer-term climate variabilities including ENSO-modoki. We note, however, that the model ENSO is weaker than real ENSO, allowing other modes to become important. With less ENSO interference, the model ENSO-modoki becomes more apparent than in nature. As the nature of ENSO-modoki is still not well known, further investigation of this phenomenon will also deepen our understanding of the role of climate variabilities in the global carbon cycle. While this study has focused on the most dominant climate variabilities in the tropical Pacific, we must note that other climate variabilities in higher latitudes, such as the North Atlantic Oscillation or Southern Ocean Annular Mode, likely play certain roles in the global carbon cycle. Although we use a complex climate-carbon cycle coupled model, our experiment is a simple and idealized case where anthropogenic CO2 emissions are not imposed. Since the ENSO and ENSO-like variabilities are changing in the present global warming trend, experiments with CO2 forcing under multiple scenarios is an area of future work. Efforts to continue observations of global scale CO2 distribution are also highly desirable in order to enable data analysis on the relations between long-term climate and carbon cycle variabilities.

## Competing interests

The authors declare that they have no competing interests.

## Authors' contributions

Both authors equally contributed to the paper and approved the final manuscript.
